# Eight-and-a-Half Syndrome: An Uncommon but Potentially Life-Threatening Disease Secondary to Acute Brainstem Infarction

**DOI:** 10.7759/cureus.86360

**Published:** 2025-06-19

**Authors:** Nicholsan Jesiah, Mayurathan P

**Affiliations:** 1 University Medical Unit, Teaching Hospital Batticaloa, Batticaloa, LKA; 2 Department of Clinical Science, Teaching Hospital Batticaloa, Batticaloa, LKA

**Keywords:** eight-and-a-half syndrome, internuclear ophthalmoplegia (ino), magnetic resonance imaging ( mri), medial longitudinal fasciculus (mlf), paramedian pontine reticular formation (pprf)

## Abstract

A localized pontine lesion affecting the abducens nucleus or paramedian pontine reticular formation (PPRF), the medial longitudinal fasciculus (MLF), and the facial nerve fascicle results in eight-and-a-half syndrome, a rare neurological condition. It manifests as ipsilateral peripheral facial nerve palsy, internuclear ophthalmoplegia (INO), and horizontal gaze palsy. This condition typically indicates a minor but well-placed infarct in the dorsal pons. We diagnosed eight-and-a-half syndrome in a patient who presented with dizziness and double vision, with a background history of hypertension and type 2 diabetes mellitus. A neurological examination showed right-sided lower motor neuron face paralysis, INO in the contralateral eye, and horizontal gaze palsy to the right, all of which are indicative of eight-and-a-half syndrome. An acute ischemic infarct was seen on magnetic resonance imaging (MRI) in the right posterior pons, which is next to the fourth ventricle. The patient started on a high-dose statin, dual antiplatelet therapy, and supportive care. His symptoms gradually improved over the next few weeks. The clinical significance of eight-and-a-half syndrome as a sign of localized pontine infarction is demonstrated by this instance. Neuroimaging and early detection are crucial for diagnosis and prompt stroke treatment.

## Introduction

A localized lesion in the dorsal pons causes the uncommon neuro-ophthalmological disorder known as eight-and-a-half syndrome. The syndrome was initially identified by Eggenberger in 1998 and is typified by ipsilateral lower motor neuron facial nerve palsy and one-and-a-half syndrome, which includes internuclear ophthalmoplegia (INO) and conjugate horizontal gaze palsy [1,2]. The lesion affects the medial longitudinal fasciculus (MLF), the facial nerve fascicle, and the abducens nucleus or paramedian pontine reticular formation (PPRF) on the same side of the brainstem [3]. Though demyelinating disorders and space-occupying lesions have also been linked to the condition, ischemic stroke is the most frequent cause [4].

Clinically, patients exhibit facial asymmetry and diplopia, which limits horizontal sight. A modest, well-placed pontine infarct is frequently the cause of eight-and-a-half syndrome despite its striking clinical appearance. Accurate localization, timely imaging, and timely therapy of underlying etiologies, such as ischemic stroke, depend on early detection of this illness.

## Case presentation

A 67-year-old man who is right-handed and has a history of hypertension and type 2 diabetes mellitus arrived at the emergency department complaining of a sudden onset of dizziness and double vision. The symptoms developed gradually and continued to exist with little variation. He denied experiencing loss of consciousness, numbness, dysarthria, dysphagia, or limb weakness. There was no history of fever or recent trauma.

**Figure 1:**
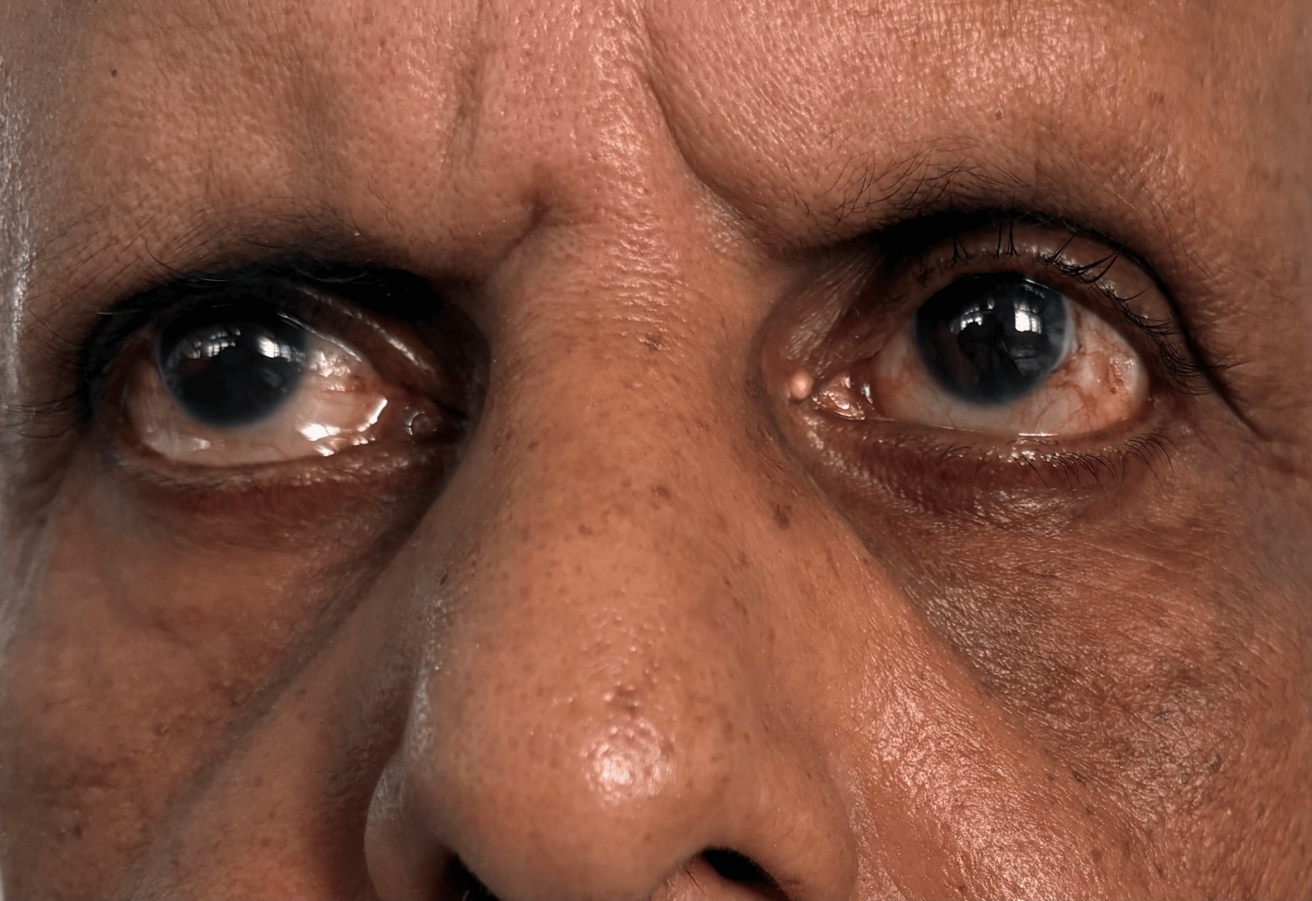
Right horizontal gaze palsy

**Figure 2:**
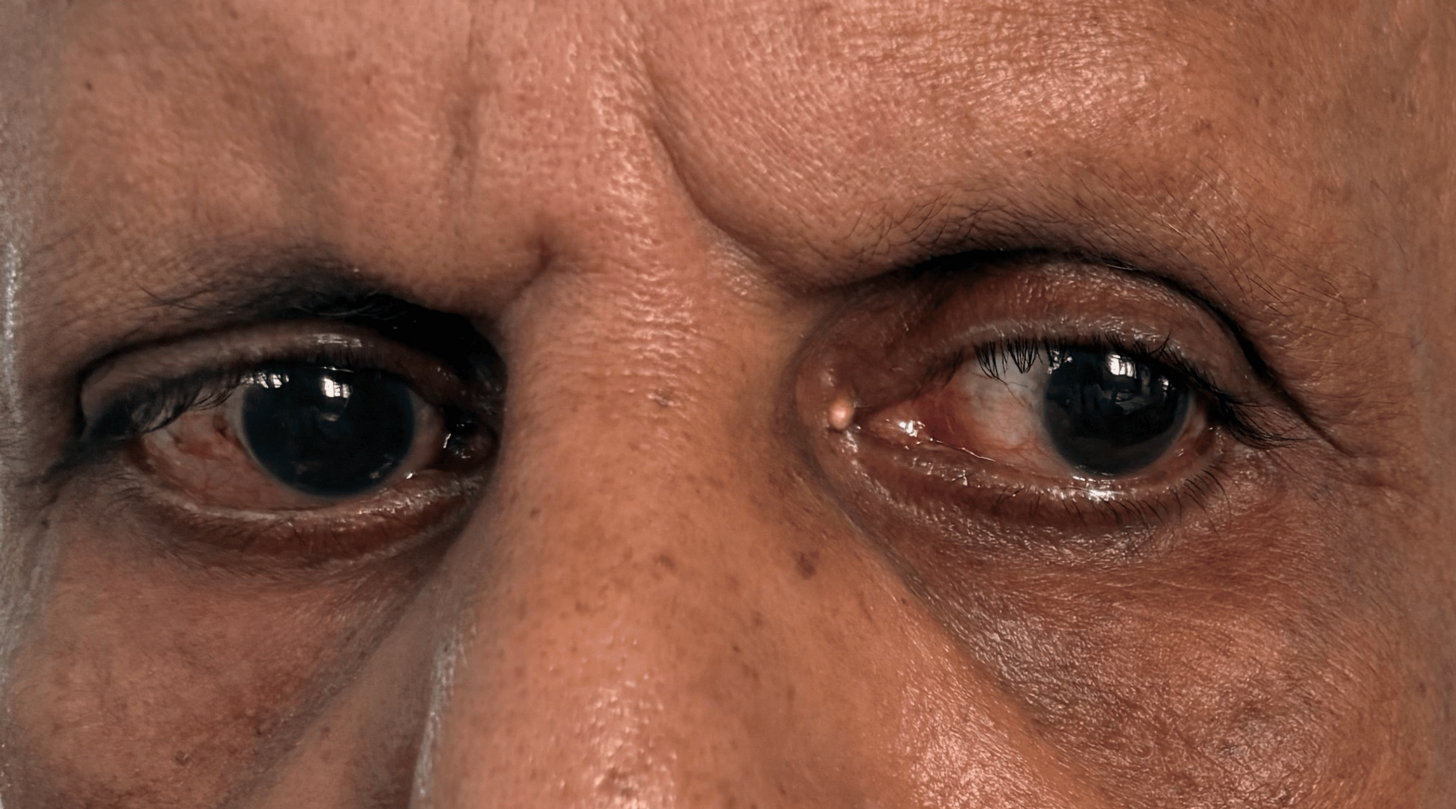
Decreased adduction of the right eye

**Figure 3:**
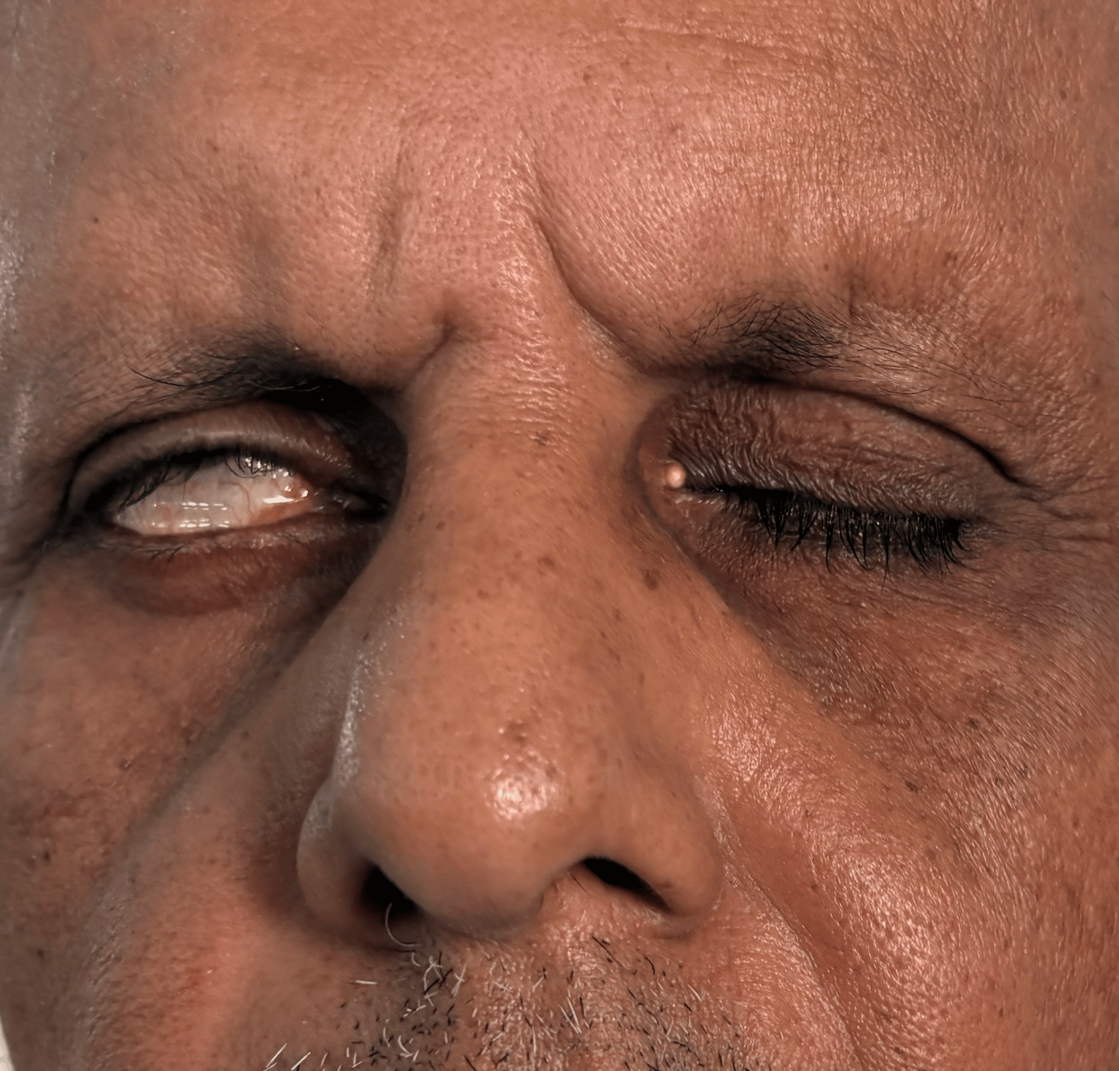
Lower motor neuron type of right side seventh cranial nerve (facial nerve) palsy: right side partial eye closure

**Figure 4:**
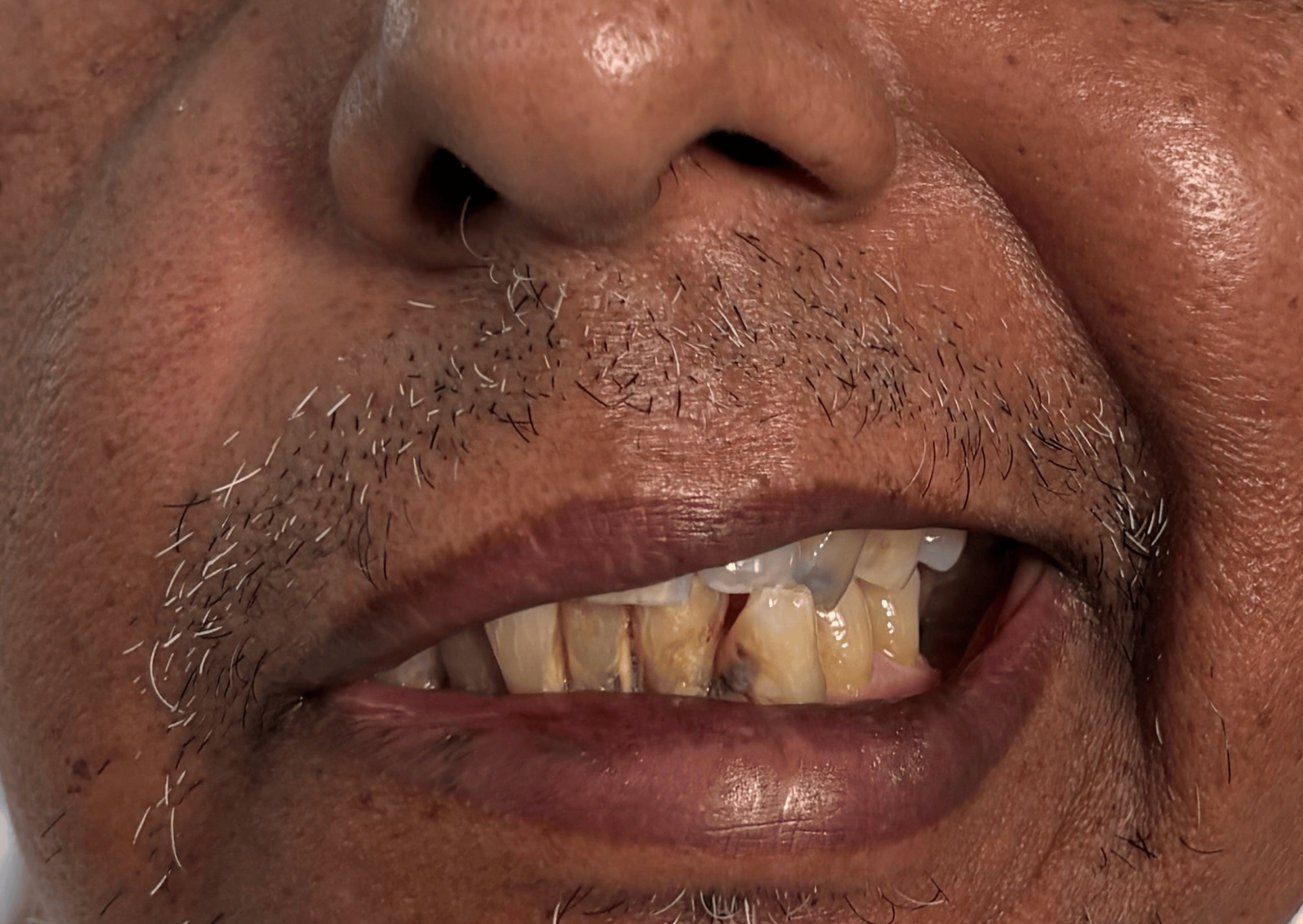
Lower motor neuron type of seventh cranial nerve (facial nerve) palsy: asymmetry of the nasolabial fold

**Figure 5:**
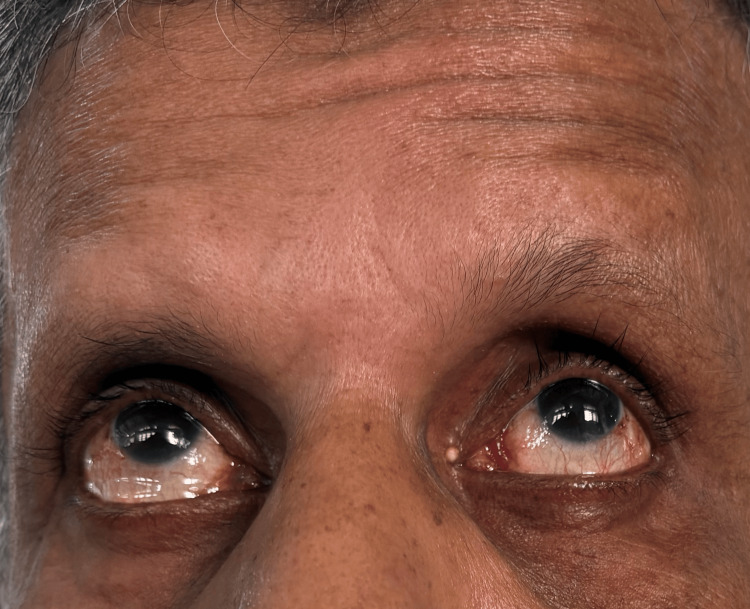
Lower motor neuron type of seventh cranial nerve (facial nerve) palsy: inability to raise the right eyebrow

A neurological evaluation revealed that the patient was focused and awake. Conjugate gaze palsy to the right (Figure 1), with decreased adduction of the right eye (Figure 2) and nystagmus of the abducting left eye on leftward gaze, was noted on cranial nerve assessment. These findings are consistent with INO. There was also a right-sided facial palsy of the lower motor neuron type, which was characterized by partial eye closure (Figure 3), asymmetry of the nasolabial fold (Figure 4), and an inability to raise the right eyebrow (Figure 5). Both pupillary responses and vertical gazing were maintained. All limbs' motor and sensory examinations were found to be normal, and cerebellar testing showed normal findings.

**Figure 6:**
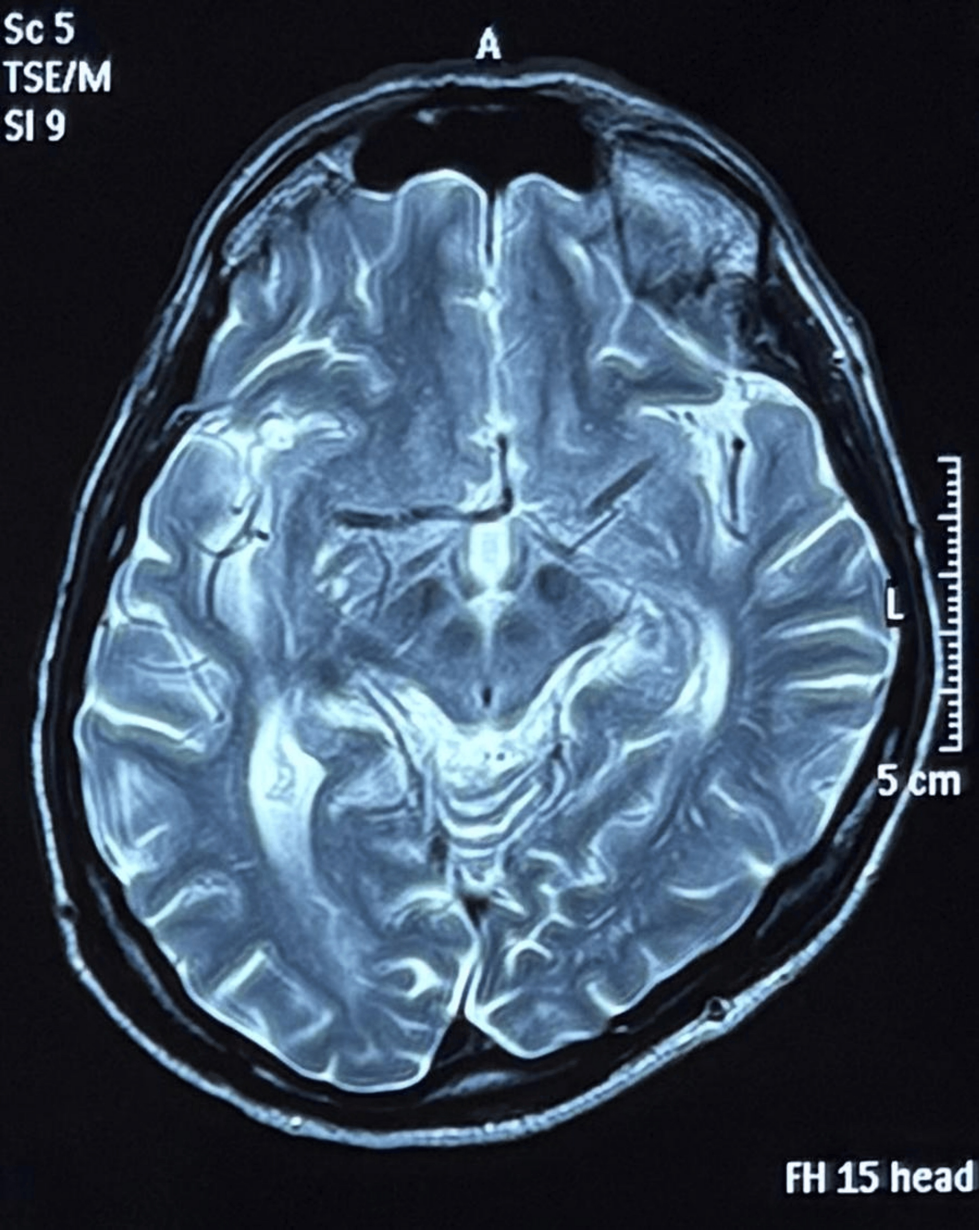
T2-weighted (T2W) magnetic resonance imaging (MRI) showed hyperintense lesion at the level of right pons

The brain's posterior part of the right pons, which is next to the fourth ventricle, has an acute ischemic infarct (Figure 6), according to magnetic resonance imaging (MRI). The diagnosis of eight-and-a-half syndrome, which is caused by a modest, carefully positioned pontine infarct, was validated by the clinical and radiological results.

A high-intensity statin (oral Atorvastatin 40 mg nocte), dual antiplatelet medication (oral Aspirin 75 mg nocte and oral Clopidogrel 75 mg nocte), and the best possible management of his vascular risk factors were used to treat the patient as though he had suffered a mild ischemic stroke. He was regularly observed and rehabilitated. His facial weakness showed some improvement during the two-week follow-up, but the deficiencies in horizontal gaze remained.

The patient has consented to using his photographs for publication and a signed consent statement from the patient has been received by the authors.

## Discussion

A localized lesion in the pontine tegmentum causes eight-and-a-half syndrome, a rare neuro-ophthalmologic condition that affects three important structures, which are MLF, PPRF, also known as the abducens nucleus, and the adjacent facial nerve fascicle [5]. This combination results in a unique clinical triad: ipsilateral peripheral facial nerve palsy, INO, and horizontal gaze palsy on the same side. The syndrome is appropriately called "eight and a half" since it is the result of combining the symptoms of one-and-a-half syndrome (horizontal gaze palsy+INO) and a seventh cranial nerve palsy [6].

INO, a lower motor neuron facial palsy on the same side, and conjugate gaze palsy to the right were among the characteristic clinical symptoms of the illness that our patient displayed. An acute infarction that was localized to the posterior pons next to the fourth ventricle - anatomically equivalent to the area that contains the MLF, facial nerve fascicle, and PPRF/abducens nucleus - was confirmed by MRI.

Although the most frequent cause is vascular lesions, other causes of tiny pontine infarcts brought on by the blockage of perforating arteries from the basilar artery, demyelinating disorders (like multiple sclerosis), space-occupying lesions, and brainstem encephalitis may also be involved [7]. In our instance, the patient was probably susceptible to small vessel ischemic disease, which resulted in an infarct, due to long-standing diabetes mellitus and hypertension.

Antiplatelet therapy, statins, and stringent glycemic and blood pressure control are the mainstays of supportive care, which aims to reduce further vascular risk. Depending on the degree of neuronal injury, recovery is frequently just partial. Our patient's facial palsy partially resolved, and their eye movement abnormalities stabilized as a result of early detection and the start of stroke care methods.

This instance highlights how crucial it is to identify complex brainstem diseases by in-depth neurological evaluation, particularly in environments with limited resources. Despite being uncommon, eight-and-a-half syndrome is a remarkable clinical entity that serves as an example of the accurate neuroanatomical localization that can be achieved by bedside examination. To direct the right course of treatment, stop progression, and maximize recovery results, early identification is crucial.

## Conclusions

A rare but clinically unique pontine stroke disease, eight-and-a-half syndrome is characterized by accurate neuroanatomical localization, including the facial nerve fascicle, MLF, and PPRF or abducens nucleus. Because it allows for quick diagnosis and the implementation of stroke management techniques, early detection based on a thorough neurological examination is essential. This example emphasizes how crucial it is to rule out brainstem infarcts in patients who exhibit unilateral facial paralysis, diplopia, and dizziness, particularly in those who have vascular risk factors, including diabetes mellitus and hypertension. Understanding these syndromes helps to maximize therapeutic outcomes by early intervention and secondary prevention, in addition to increasing diagnostic accuracy. This case highlights another potential for a significant recovery in patients with eight-and-a-half syndrome when timely and appropriate treatment is administered.
